# A comparative study of curated contents by knowledge-based curation system in cancer clinical sequencing

**DOI:** 10.1038/s41598-019-47673-9

**Published:** 2019-08-05

**Authors:** Kazuko Sakai, Masayuki Takeda, Shigeki Shimizu, Takayuki Takahama, Takeshi Yoshida, Satomi Watanabe, Tsutomu Iwasa, Kimio Yonesaka, Shinichiro Suzuki, Hidetoshi Hayashi, Hisato Kawakami, Yoshikane Nonagase, Kaoru Tanaka, Junji Tsurutani, Kazumasa Saigoh, Akihiko Ito, Tetsuya Mitsudomi, Kazuhiko Nakagawa, Kazuto Nishio

**Affiliations:** 1 Department of Genome Biology, Kindai University Faculty of Medicine, Osaka-Sayama, Osaka, 589-8511 Japan; 2 Department of Medical Oncology, Kindai University Faculty of Medicine, Osaka-Sayama, Osaka, 589-8511 Japan; 3 Department of Pathology, Kindai University Faculty of Medicine, Osaka-Sayama, Osaka, 589-8511 Japan; 4 Department of Clinical Genetics, Kindai University Faculty of Medicine, Osaka-Sayama, Osaka, 589-8511 Japan; 5 Department of Thoracic Surgery, Kindai University Faculty of Medicine, Osaka-Sayama, Osaka, 589-8511 Japan

**Keywords:** Cancer genomics, Targeted therapies

## Abstract

Medical oncologists are challenged to personalize medicine with scientific evidence, drug approvals, and treatment guidelines based on sequencing of clinical samples using next generation sequencer (NGS). Knowledge-based curation systems have the potential to help address this challenge. We report here the results of examining the level of evidence regarding treatment approval and clinical trials between recommendations made by Watson for Genomics (WfG), QIAGEN Clinical Insight Interpret (QCII), and Oncomine knowledge-based reporter (OKR). The tumor samples obtained from the solid cancer patients between May to June 2018 at Kindai University Hospital. The formalin-fixed paraffin-embedded tumor samples (n = 31) were sequenced using Oncomine Comprehensive Assay v3. Variants including copy number alteration and gene fusions identified by the Ion reporter software were used commonly on three curation systems. Curation process of data were provided for 25 solid cancers using three curation systems independently. Concordance and distribution of curated evidence levels of variants were analyzed. As a result of sequencing analysis, nonsynonymous mutation (n = 58), gene fusion (n = 2) or copy number variants (n = 12) were detected in 25 cases, and subsequently subjected to knowledge-based curation systems (WfG, OKR, and QCII). The number of curated information in any systems was 51/72 variants. Concordance of evidence levels was 65.3% between WfG and OKR, 56.9% between WfG and QCII, and 66.7% between OKR and QCII. WfG provided great number of clinical trials for the variants. The annotation of resistance information was also observed. Larger differences were observed in clinical trial matching which could be due to differences in the filtering process among three curation systems. This study demonstrates knowledge-based curation systems (WfG, OKR, and QCII) could be helpful tool for solid cancer treatment decision making. Difference in non-concordant evidence levels was observed between three curation systems, especially in the information of clinical trials. This point will be improved by standardized filtering procedure and enriched database of clinical trials in Japan.

## Introduction

Medical oncologists are challenged to personalize medicine with rapidly changing scientific evidence, drug approvals, and treatment guidelines. Next generation sequencer (NGS)-based clinical sequencing is approved as *in vitro* medical diagnostics (IVD) and companion diagnostics for cancer patients.

Clinical sequencing reports are a key component of the clinical sequencing process and in Japan, these are prepared by an expert panel (clinical sequencing team) in each individual institute^1^. Curation of the annotated gene variants is performed manually by the members of expert panel and is supported by several knowledgebases^2–5^. However, the manual curation procedure in the expert panel is time consuming and variation of curation contents often occurs. Efforts to standardize the clinical sequencing report have been undertaken by various Japan cancer related associations and have provided guidance for cancer clinical sequencing^6^. However, as a result recent technological advances, practical clinical approaches capable of handling robust curated data with automated clinical annotation for the clinician is required. In Japan, the C-CAT (Center for Cancer Genomics and Advanced Therapeutics) database and curation systems has been under construction, and this system operation has started. Gene panel testing that is accompanied by curation results like FoundationOne CDx is uncommon. In addition, the status of drug approval and clinical trials are different among countries.

Manual curation by institutional molecular tumor board is time-consuming, cumbersome and relatively difficult to update in real-time. Information retrieval is an area of expertise in computing and knowledge-based curation systems have the potential to improve and facilitate genetic testing^7,8^. Furthermore, implementing a global approach will help create and standardized clinical sequencing reporting enabling clinicians to better select therapies for cancer patients and match patients to appropriate clinical trials.

Currently several approaches and are being developed and implemented in genetic testing^7–9^. Herein, we compare three global curation systems; Watson for Genomics (WfG, IBM), Oncomine knowledge-based reporter (OKR, Thermo Fisher Scientific) and QIAGEN Clinical Insight Interpret (QCII, Qiagen) in a cohort of Japanese cancer patients and evaluate them for efficiency in reporting, therapy selection based on levels of evidence, and clinical trial matching.

## Results

### Targeted sequencing and annotation

**Figure 1:**
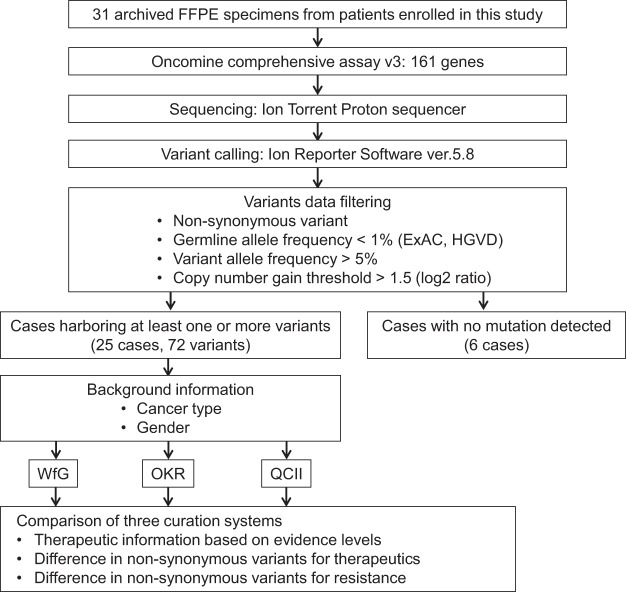
CONSORT diagram for the study. FFPE specimens obtained from 31 solid tumor patients were subjected to Oncomine Comprehensive Assay v3. Data of variant calling as non-synonymous mutations and copy number variants by Ion reporter were filtered out based on following cut off values; quality score >100, germline allele frequency <1% (ExAC, HGVD), variant allele frequency >5%, and copy number gain threshold >1.5 (log2 ratio). Calling data of fusion transcripts were uploaded unfiltered. Variant data of 25 cases in which at least one variant was detected was analyzed by WfG, OKR, and QCII. Cancer type and gender of the patients, in addition to the filtered data, were uploaded to the curation systems.

Tumor tissue samples were obtained from the patients with various types of solid cancers were performed using OCAv3, and annotated treatment information by three knowledge systems (Fig. 1**)**. The age of enrolled 31 patients (male 16, female 15) was 10–70’s. Types of cancer are shown in Table 1. The samples were obtained before drug therapy except for one case. Tissue specimen of the exceptional case were obtained at progressive disease to molecularly targeted therapy. The most frequent cancer type was lung cancer. All samples were sequenced successfully. The average depth was ranged from 373 to 2206 in 31 cases. Analyses of mutations, fusions, and copy number alterations were performed using the Ion Reporter software (ver.5.8). Nonsynonymous variants (n = 58), copy number gain (n = 12), or gene fusions (n = 2) were detected in 25/31 (80.6%) cases. The average number of variants in the 25 cases were 2.8 (1 to 7). The frequently called variants was *TP53* mutation which was detected in 12 tumor samples.

**Table 1:** Type of cancer.

Cancer type	Number of pts
Lung cancer	14
Breast cancer	2
Gastric cancer	2
Osteosarcoma	2
Breast cancer (TNBC)	1
Liposarcoma	1
Tongue cancer	1
Colorectal cancer	1
Endometrial cancer	1
Prostate cancer	1
Cervical cancer	1
Leiomyosarcoma	1
Unknown primary cancer	1
Sweat gland tumor	1
Ovarian cancer	1

### Curated contents by three curation systems

The results were compiled by three knowledge-based report systems. The vcf files of 25 case sequencing data with one or more nonsynonymous variants were curated using WfG, OKR, and QCII. The curated contents of the report were classified; Evidence levels of curated contents of pathogenic (or likely pathogenic) variants were classified as (i) approved for the said cancer type (level I), (ii) approved for other cancer type (level II), (iii) clinical trials (level III), (iv) none (Supplementary Table 1). Regarding clinical trials, WfG, OKR, QCII annotated clinical studies in any countries, Asian countries (Japan, Korea, China, Taiwan, and Singapore), and Japan, respectively. Number of evidence level in three curation systems was counted. Curated contents on variant unknown significance (VUS) and gene alteration designated as benign or likely benign in the report was not counted.

**Figure 2:**
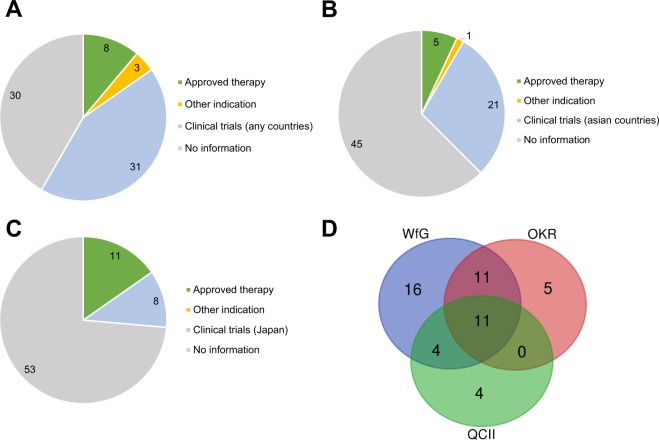
Distribution of evidence level for treatment choice and clinical trials reported by curation systems. (**A**) The pie chart shows the distribution of levels of the curated therapeutic information curated by WfG. Green; approved for the said cancer type (evidence level 1), orange; approved for other cancer type (evidence level II), blue; information of clinical trials in any countries (level III). (**B**) The pie chart shows the distribution of levels of the curated therapeutic information curated by OKR. Green; approved for the said cancer type (level 1), orange; approved for other cancer type (evidence level II), blue; information of clinical trials in Asian countries (Japan, Korea, China, Taiwan, Singapore) (level III). (**C**) The pie chart shows the distribution of levels of the curated therapeutic information curated by QCII. Green; approved for the said cancer type (level 1), orange; approved for other cancer type (level II), blue; information of clinical trials in Japan (level III). (**D**) Venn diagram shows the commonality of clinically informative genetic variants (across all evidence levels, see Supplementary Table 1) between curation systems.

WfG annotated therapeutic information in 8/72 (11.1%) as level I, 3/72 (4.2%) as level II, and 31/72 (43.1%) as possible clinical trials (Fig. 2A). OKR annotated therapeutic information in 5/72 (6.9%) as level I, 1/72 (1.4%) as level II, and 21/72 (29.2%) as level III as possible clinical trials (Fig. 2B). QCII annotated therapeutic information in 11/72 (15.3%) as level I and 8/72 (11.1%) as level III (Fig. 2C). *EGFR* mutation and *EML4-ALK* fusion gene for lung cancer were common variants described in three systems with the same evidence levels (level I). Different evidence level was described in 46/72 variants among three systems (Fig. 2D). The number of genes described by one system only was 5 for WfG (*ATM* mutation, *CREBBP* mutation, *TP53* mutation, *CCND1* amp, *CCNE1* amp), 4 for OKR (*SLX4* mutation, *FANCl* mutation, *SETD2* mutation, *ERBB3* mutation), and 4 for QCII (*BRCA2* mutation, *PDGFRA* mutation, *CDK6* mutation, *ESR1* amp). Druggable alterations were annotated in 11 variants by WfG, OKR, and QCII systems. In particular, *EGFR* ex19 and *EML4-ALK* fusion transcript were annotated in 4 cases. These gene alterations were recognized as actionable changes for the non-small cell lung cancer and classified as level I in most of the world. Four called variants (4/11, 36.3%) were classified into level I were common in all systems. The remaining 7 variants were classified into different evidence levels between WfG, OKR, and QCII systems. These variants are *EGFR* and *MET* copy number gains, and *PIK3CA*, *ERBB2*, *KRAS* and *TSC1* non-synonymous mutations. On the other hand, actionable alterations were annotated in 15 variants by any of two systems. Only one variant was classified into class I (1/15, 6.7%) and 25 variants were annotated by only of the systems and were classified into class III. These results suggest that the variants with high evidence levels (level I) were commonly annotated in all curation systems.

We compared all annotated therapeutic information between three knowledge systems for 72 variants in 25 cases. The rate of concordance of annotated information of three systems were 65.3% (κ = 0.33, 95% CI 0.32–0.35) for WfG and OKR, 56.9% (κ = 0.21, 95% CI 0.19–0.22) for WfG and QCII, and 66.7% (κ = 0.24, 95% CI 0.23–0.26) for OKR and QCII (Table 2). When focusing on level I and II, the concordance of annotated information of three systems were 87.5% (κ = 0.41, 95% CI 0.37–0.44) for WfG and OKR, 86.1% (κ = 0.46, 95% CI 0.43–0.50) for WfG and QCII, and 93.1% (κ = 0.67, 95% CI 0.62–0.72) for OKR and QCII (Table 3). Increased concordance rate was observed in the annotation of higher levels. In other words, the information of clinical trials was varied between three systems.

**Table 2:** Correlation of all annotated therapeutic information.

Correlation between WfG and OKR					
		OKR			Total
		Annotated		No information	
WfG	Annotated	22		20	42
	No information	5		25	30
	Total	27		45	72
Correlation between WfG and QCII					
		QCII			Total
		Annotated		No information	
WfG	Annotated	15		27	42
	No information	4		26	30
	Total	19		53	72
Correlation between OKR and QCII					
		QCII			Total
		Annotated		No information	
OKR	Annotated	11		16	27
	No information	8		37	45
	Total	19		53	72

**Table 3:** Correlation of level I and II therapeutic information.

Correlation between WfG and OKR				
		OKR		Total
		Annotated	No information	
WfG	Annotated	4	7	11
	No information	2	59	61
	Total	6	66	72
Correlation between WfG and QCII				
		QCII		Total
		Annotated	No information	
WfG	Annotated	6	5	11
	No information	5	56	61
	Total	11	61	72
Correlation between OKR and QCII				
		QCII		Total
		Annotated	No information	
OKR	Annotated	6	0	6
	No information	5	61	66
	Total	11	61	72

### Difference in curated contents between mutation, copy number alteration, and gene fusion

To know whether there is difference in curated contents for types of gene alteration, we compared concordance in clinical information levels (informative or non-informative) for mutation or copy number alteration.

Concordance of informative content for non-synonymous variants was 65.5% between WfG and OKR, 58.6% between WfG and QCII, and 69.0% between OKR and QCII. Concordance of informative content for copy number alteration was 58.3% between WfG and OKR, 41.7% between WfG and QCII, and 50.0% between OKR and QCII. Rate of informative information seems to be higher in non-synonymous variants than copy number alteration. However, there is no significant difference in three systems (data not shown).

### Curated contents of resistance information

Resistance information was defined in OKR and QCII but not WfG. In 30 out of 31 cases, the details from curated data indicated intrinsic (not acquired) resistance. In one case (S02), tumor specimens were obtained after disease progression on an ALK tyrosine kinase inhibitor. *EGFR* mutation, *KRAS* mutation, and *EML4-ALK* in lung cancer case were commonly annotated resistance information by OKR and QCII. *KRAS* mutation in endometrial cancer and *NF1* mutation in lung cancer case were annotated resistance information only in the OKR system. *KIT* amp, *EGFR* amp, *MET* amp and *ALK* mutation in lung cancer, and *CCNE1* amp in breast cancer were curated as resistant information only in the QCII. The rate of concordance of curated resistance information between OKR and QCII were 90.3% (κ = 0.58, 95% CI 0.54–0.62) (Table 4).

**Table 4:** Concordance of resistance information between OKR and QCII.

		QCII		Total
		Annotated	No information	
OKR	Annotated	6	2	8
	No information	5	59	64
	Total	11	61	72

## Discussion

In this study, we compared different clinically reporting methods using data obtained in real world clinical practice. The panel was used consisted of 161 genes selected to provide a “comprehensive” overview of data across different solid cancers. Variant data from non-synonymous mutations, copy number gains, and fusion transcripts called by Ion Reporter proceeded through a filtering process in which the final number of called variants per patient passed through and were processed by three state of the art knowledge-based curation systems. From this, actionable information, including clinical trial information was harvested and compared between three curation systems. It is clinically important evidence that clinically relevant variants (level I etc.) was detected uniformly by three curators (so it is safe, it is not missing). On the other hand, the overall concordance of evidence levels of three curation systems are relatively low (56.9%~66.7%), although lack of concordance does not necessarily provide evidence regarding which is ‘correct’ in its recommendation. There is no ‘gold standard’ beyond expert panel. In addition, there was no significant difference for clinical information rate between non-synonymous variants and copy number alteration. We did not calculate it for gene fusions, because number of gene fusion was small. Recently, the highly concordance between in house report and Watson for Oncology for breast cancer patients^10^. It will be necessary to assess concordance of curation contents between in house expert panel and curation systems in the next study.

WfG reported the evidence levels of the greater numbers of the variants^7,11^. In the curation process to inform the clinical trials, WfG and QCII used the global database. WfG was reported clinical trials without limiting the regions. QCII filtered the data to be limited to the Asian region. OKR used Asian database. WfG presented many US clinical trials (Fig. 1). The discrepancy between three systems is likely to be due to difference in the filtering procedure of regional clinical trials. In fact, when excluding the data of clinical trials (level III), they showed higher concordance rate (86.1%~93.1% for levels I and II) in three systems. It is necessary to discuss with physicians in Asian countries including Japan whether it is necessary to inform clinical trials in US and European regions. The template of OKR can be created in several languages including Japanese, Chinese, and Korean. This approach is convenience for physicians in Asian. It can be also considered to create the reports in the Asian languages in other systems.

Although the sample of this study is small, it is still adequate since purpose of this study was not to analyze statistically significant of the differences between the panels, but rather it aimed to compare the implementation of different clinically reporting methods using data obtained in real world clinical practice. In addition, actionable information, including clinical trial information, was compared between three curation systems. Actionable information for one or several variants was provided for each case and only limited information was available for use for comparative analysis. Thus for the purpose of comparing the output results of annotated alterations with regards to therapeutic indication, based on evidence level, and clinical trial matching, we considered the sample size to be adequate. Information management challenges in cancer care are occurring in a practice environment where there is little time available for tracking and accessing relevant information at the point of care^12,13^. For example, a study that surveyed 1,117 oncologists reported that on average 4.6 h per week were spent keeping current in the field; while 53 h per week were spent on patient care and administrative tasks^14^. The situation is same in Japan. Importantly, it took ten minutes or shorter to complete the curation from uploading the vcf files to getting the report using any curation system.

## Methods

### Clinical specimens

A total of 31 solid tumor patients enrolled to this study between May to June 2018 at Kindai University Hospital. All patients provided written informed consent to participation in the study, including the collection of tumor tissue for analysis. This study was conducted in compliance with the Helsinki Declaration and the Ethical Guidelines for Medical and Health Research Involving Human Subjects by the Japanese government and has been approved by the ethics committee of Kindai University Faculty of Medicine (approved no 29–203).

### Tissue processing

The collected formalin-fixed, paraffin-embedded tumor specimens underwent histological review, and only those containing sufficient tumor cells (at least 10%) as revealed by hematoxylin-eosin staining were subjected to nucleic acid extraction. DNA and RNA were isolated from the tissue with the use of an AllPrep DNA/RNA Mini Kit (Qiagen, Valencia, CA). The quality and quantity of the nucleic acid were verified with the use of a NanoDrop 2000 device, PicoGreen dsDNA Reagent, and RiboGreen RNA Reagent (all from Thermo Scientific, Wilmington, DE).

### Next-generation sequencing

Tumor DNA and RNA were subjected to analysis with Oncomine Comprehensive assay v3 (OCAv3) for detection of mutations, copy number gain, and gene fusions. For DNA library preparation, tumor DNA (20 ng) was subjected to multiplex polymerase chain reaction (PCR) amplification with the use of an Ion AmpliSeq Library Kit 2.0 and DNA OCAv3 (all from Thermo Fisher Scientific). The PCR products were ligated to Ion Xpress Barcode Adapters (Thermo Fisher Scientific) and purified with the use of Agencourt AMPure XP beads (Beckman Coulter, Brea, CA). The purified libraries were pooled and then sequenced with an Ion Torrent Proton instrument, Ion PI Hi-Q Chef Kit, and Ion PI v3 Chip (all from Thermo Fisher Scientific). DNA sequencing data was accessed through the Torrent Suite ver.5.8 program (Thermo Fisher Scientific). Reads were aligned with the hg19 human reference genome, and potential mutations and copy number alteration were called with the use of Ion Reporter™ Software ver.5.8. Raw variant calls were filtered with quality score of <100, and were manually checked using the integrative genomics viewer (IGV; Broad Institute, Cambridge, MA). Germline mutations were excluded with the use of the Human Genetic Variation Database (http://www.genome.med.kyoto-u.ac.jp/SnpDB)^15^ and Exome Aggregation Consortium database (http://exac.broadinstitute.org/). For RNA library preparation, tumor RNA (20 ng) was subjected to reverse transcription with the use of a SuperScript IV VILO Master Mix (Thermo Fisher Scientific) followed by library generation with the use of an Ion AmpliSeq Library Kit 2.0 and RNA OCAv3 (all from Thermo Fisher Scientific). The PCR products were ligated to Ion Xpress Barcode Adapters (Thermo Fisher Scientific) and purified with the use of Agencourt AMPure XP beads (Beckman Coulter, Brea, CA). The purified libraries were pooled and then sequenced with an Ion Torrent Proton instrument, Ion PI Hi-Q Chef Kit, and Ion PI v3 Chip (all from Thermo Fisher Scientific). RNA sequencing data were accessed through the Torrent Suite ver.5.8 program (Thermo Fisher Scientific). Reads were aligned with the hg19 human reference genome, and potential fusions were analyzed with the use of Ion Reporter™ Software ver.5.8.

### Watson for genomics

The following information was uploaded to Watson for Genomics (WfG): cancer type, a list of variants as a variant calling file, copy number alterations as linear copy-number values file, and fusion status as gene name (Supplementary Table 2). After the above information was uploaded, the WfG returns detailed annotation of 1) mutation profile (benign, likely benign, likely pathogenic, pathogenic, and variant of unknown significance, VUS) and 2) drug (evidence level, approval status in U.S. Food and Drug Administration, clinical trials, potential therapeutic options, and literature). Benign and likely benign variants were removed from the report (Supplementary Table 3). A report was generated by WfG showing the variants alongside potential targeted drugs. An example of the interface is shown in supplemental data. Software version was V38.159 (01-JUN-2018) and V39.135 (20-JUL-2018) in this study.

### Oncomine knowledgebase reporter

The vcf files generated by Ion Reporter™ Software with the information of cancer type was uploaded to Oncomine Knowledgebase Reporter (OKR) (Supplementary Table 2). After the above information was uploaded, the OKR returns detailed annotation of 1) mutation profile (level of evidence based on a Joint Consensus Recommendation in ASCO, AMP, and CAP)^16^ and 2) drug (approval status in U.S. Food and Drug Administration, clinical trials, potential therapeutic options, and literature) (Supplementary Table 3). The location of clinical trial was set at Japan, Korea, and China. A report was generated by OKR showing the variants alongside potential targeted drugs. An example of the interface is shown in supplemental data. Software version was V3.2 (2018.03 (006)) and V3.3 (2018.06(004)) in this study.

### QIAGEN clinical insight interpret

The vcf files generated by Ion Reporter™ Software with the information of cancer type was uploaded to QIAGEN Clinical Insight Interpret (QCII) (Supplementary Table 2). After the above information was uploaded, the QCII returns detailed annotation of 1) mutation profile (computed classification according to previous recommendation)^16^ and 2) drug (phase in U.S. Food and Drug Administration, clinical trials, potential therapeutic options, and literature) (Supplementary Table 3). The location of clinical trial was set at Japan. A report was generated by QCII showing the variants alongside potential targeted drugs. An example of the interface is shown in supplemental data. Software was used from 19^th^ June 2018 to 31th July 2018 in this study.

### Statistical analysis

The kappa statistic and associated 95% confidence intervals were used to measure agreement among curation methods.

## Conclusion

Watson for Genomics (WfG), QIAGEN Clinical Insight Interpret (QCII), and Oncomine knowledge-based reporter (OKR) could work in the NGS based clinical sequencing with expert panel in routine use for patients with solid cancers. Region-specific automatic curation algorithms are necessary in a global curation system.

## Supplementary information

Supplementary Tables 1–3
