# Supplementary material for: A comparative study of curated contents by knowledge-based curation system in cancer clinical sequencing
Source: Sci Rep. 2019 Aug 5;9:11340. doi: 10.1038/s41598-019-47673-9 (PMC6683116; doi:10.1038/s41598-019-47673-9)
Supplement: Supplementary file 1 — Supplementary Tables 1–3 [file 41598_2019_47673_MOESM1_ESM.pdf]

Supplementary information

A comparative study of curated contents by knowledge-based curation system in cancer clinical sequencing

Kazuko Sakai[1], Masayuki Takeda[2], Shigeki Shimizu[3], Takayuki Takahama[2], Takeshi Yoshida[2], Satomi Watanabe[2], Tsutomu Iwasa[2], Kimio Yonesaka[2], Shinichiro Suzuki[2], Hidetoshi Hayashi[2], Hisato Kawakami[2], Yoshikane Nonagase[2], Kaoru Tanaka[2], Junji Tsurutani[2], Kazumasa Saigoh[4], Akihiko Ito[3], Tetsuya Mitsudomi[5], Kazuhiko Nakagawa[2] and Kazuto Nishio[1]

[1]Department of Genome Biology, [2]Department of Medical Oncology, [3]Department of Pathology, [4]Department of Clinical Genetics, [5]Department of Thoracic Surgery, Kindai University Faculty of Medicine, Osaka-Sayama, Osaka 589-8511, Japan.

Correspondence and requests for materials should be addressed to K.N. (email: knishio@med.kindai.ac.jp)

Supplementary Table 1. Annotation information on the detected 72 variants in 25 cases.

ID	Variants	WfG	OKR	QCII
S21	EGFR:p.Glu746_Ala750del	I	I	I
S25	EGFR:p.Glu746_Ala750del	I	I	I
S02	EML4-ALK fusion	I	I	I
S28	EML4-ALK fusion	I	I	I
S20	EGFR CNG	I	III	I
S04	PIK3CA:p.His1047Arg	I	III	III
S20	MET CNG	III	I	I
S18	ERBB2:p.Ser310Phe	III	II	I
S01	KRAS:p.Gly12Val	III	III	I
S26	KRAS:p.Gly12Cys	III	III	I
S30	TSC1:p.Ser163*	III	III	III
S02	ALK:p.Gly1202Arg	I		I
S14	PIK3CA:p.His1047Arg	I	III	
S04	MYC CNG	III		I
S20	KIT CNG	II	III	
S29	PIK3CA CNG	II	III	
S31	PIK3CA:p.Pro449_Leu452del	II		III
S05	PIK3R1:p.Tyr431*	III	III	
S05	NF2:p.Thr59fs	III	III	
S08	NF1:p.Leu1253fs	III	III	
S11	KRAS:p.Gly12Val	III	III	
S14	FGFR2:p.Lys659Asn	III	III	
S14	AKT3 CNG	III	III	
S18	FGFR2:p.Cys382Arg	III	III	
S27	TP53:p.Glu285Lys	III	III	
S20	CDK6 CNG	III		III
S01	TP53:p.Glu204*	III		
S03	TP53:p.Cys242Tyr	III		
S03	CCND1 CNG	III		
S04	CCNE1 CNG	III		
S05	ATM:p.Gln1017*	III		
S07	TP53:p.Val172_Glu180del	III		
S08	TP53:p.Arg65*	III		
S09	TP53:p.Phe270Leu	III		
S09	CCNE1 CNG	III		
S10	TP53:p.Met237Ile	III		
S18	CREBBP:p.Arg768*	III		
S20	TP53:p.Glu294*	III		
S21	TP53:p.Arg175Gly	III		
S24	TP53:p.Arg110Pro	III		
S25	TP53:p.Asp148fs	III		
S29	TP53:p.Ser127Phe	III		
S10	FANCI:p.Gln1109*		III	
S10	FANCI:p.Gln1217*		III	
S13	ERBB3:p.Met60Arg		III	
S28	SETD2:p.Gln1343*		III	
S29	SLX4:p.Ser1228fs		III	
S07	CDK6:p.Phe164fs			III
S09	BRCA2:p.Arg1160Thr			III
S16	PDGFRA:p.Asp784Gly			III
S20	ESR1 CNG			III
S04	SLX4:p.Ala952Met			
S05	ARID1A:p.Met737Val			
S05	NF1:p.Val2050Phe			
S08	SMARCA4:p.Leu857_Tyr860del			
S09	CREBBP:p.Gln2300His			
S10	NF1:p.Arg1132His			
S10	STK11:p.Arg106Trp			
S11	FLT3:Val512Leu			
S11	TSC2:Arg1174Gln			
S13	ARID1A:p.Pro2114Ser			
S14	MLH1:p.Glu430Lys			
S19	PPARG:p.Arg403His			
S20	SMARCA4:p.Glu1252*			
S24	FBXW7:p.Asn633Ser			
S24	FANCA:p.Tyr579Cys			
S25	STK11:p.Ile46Met			
S25	MDM4 CNG			
S27	FANCD2:p.Ser996Gly			
S29	ATM:p.Leu75Met			
S30	NOTCH2:p.Gly292*			
S31	CHEK1:p.Asp434His			

I, approved for the said cancer type; II, approved for other cancer type; III, information of clinical trials

Legend:
- Evidence level I
- Evidence level II
- Evidence level III
- No therapeutic information

Supplementary Table 2. Data format for input.

Types of gene alteration	Panel		
	WfG	QCII	OKR
Mutation/Indel	.vcf format	.vcf format	.vcf format
Fusion	.fusion format	direct input	.vcf format
Copy number variation	.log2 format	direct input	.vcf format

Supplementary Table 3. Information of output.

	Variant Effect Predictor	Evidence level for therapeutic information	Evidence level for this report
WfG	Actionable	Level 1-4, R1-2	Level I, II, III*
QCII	Pathogenic/likely pathogenic/Uncertain significance	Level 1A - 3	
OKR	-	Level IA - IIC	

*I, approved for the said cancer type; II, approved for other cancer type; III, information of clinical trials.
